# Small extracellular vesicles derived from hair follicle neural crest stem cells enhance perineurial cell proliferation and migration via the TGF-β/SMAD/HAS2 pathway

**DOI:** 10.4103/NRR.NRR-D-25-00127

**Published:** 2025-07-25

**Authors:** Yiming Huo, Bing Xiao, Haojie Yu, Yang Xu, Jiachen Zheng, Chao Huang, Ling Wang, Haiyan Lin, Jiajun Xu, Pengfei Yang, Fang Liu

**Affiliations:** 1 Department of Human Anatomy, Naval Medical University, Shanghai, China; 2 Department of Orthopedic Surgery, Affiliated Changzheng Hospital, Naval Medical University, Shanghai, China; 3 Department of Special Clinic, Affiliated Changhai Hospital, Naval Medical University, Shanghai, China; 4 Neurovascular Center, Affiliated Changhai Hospital, Naval Medical University, Shanghai, China

**Keywords:** hair follicle neural crest stem cells, HAS2, migration, miR-21-5p, perineurial cells, proliferation, peripheral nerve injury, Smad7, small extracellular vesicles, transforming growth factor-β/SMAD signaling pathway

## Abstract

Peripheral nerve defect repair is a complex process that involves multiple cell types; perineurial cells play a pivotal role. Hair follicle neural crest stem cells promote perineurial cell proliferation and migration via paracrine signaling; however, their clinical applications are limited by potential risks such as tumorigenesis and xenogeneic immune rejection, which are similar to the risks associated with other stem cell transplantations. The present study therefore focuses on small extracellular vesicles derived from hair follicle neural crest stem cells, which preserve the bioactive properties of the parent cells while avoiding the transplantation-associated risks. *In vitro*, small extracellular vesicles derived from hair follicle neural crest stem cells significantly enhanced the proliferation, migration, tube formation, and barrier function of perineurial cells, and subsequently upregulated the expression of tight junction proteins. Furthermore, in a rat model of sciatic nerve defects bridged with silicon tubes, treatment with small extracellular vesicles derived from hair follicle neural crest stem cells resulted in higher tight junction protein expression in perineurial cells, thus facilitating neural tissue regeneration. At 10 weeks post-surgery, rats treated with small extracellular vesicles derived from hair follicle neural crest stem cells exhibited improved nerve function recovery and reduced muscle atrophy. Transcriptomic and microRNA analyses revealed that small extracellular vesicles derived from hair follicle neural crest stem cells deliver miR-21-5p, which inhibits mothers against decapentaplegic homolog 7 expression, thereby activating the transforming growth factor-β/mothers against decapentaplegic homolog signaling pathway and upregulating hyaluronan synthase 2 expression, and further enhancing tight junction protein expression. Together, our findings indicate that small extracellular vesicles derived from hair follicle neural crest stem cells promote the proliferation, migration, and tight junction protein formation of perineurial cells. These results provide new insights into peripheral nerve regeneration from the perspective of perineurial cells, and present a novel approach for the clinical treatment of peripheral nerve defects.

## Graphical abstract

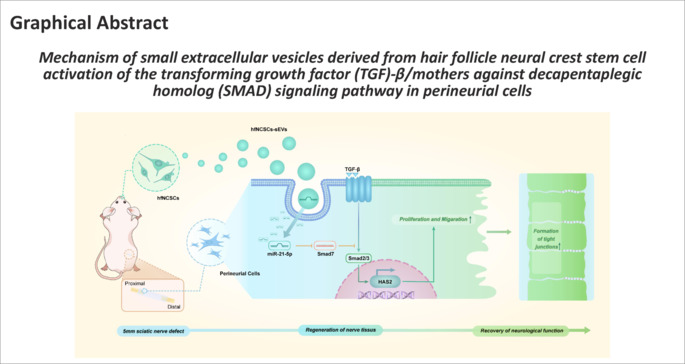

## Introduction

Peripheral nerve injury remains a prevalent clinical challenge. Among these injuries, peripheral nerve defects often lead to impairments in motor, sensory, and autonomic nerve functions, significantly reducing quality of life (Sarhane et al., 2023; Aldali et al., 2025). The processes of tissue regeneration and repair following peripheral nerve defects are complex and involve various cell types. Previous studies have primarily focused on Schwann cells and macrophages. However, recent studies have indicated that perineurial cells (PCs) may also play a crucial role in this process (Carr et al., 2019; Chovatiya et al., 2023; Zhao et al., 2024). Currently, there are numerous theories regarding the origin and characteristics of PCs, and a lack of consensus remains. A three-dimensional model of neural development was used to demonstrate that a portion of PCs may originate from neural crest stem cells (NCSCs) (Anderson et al., 2018). PCs are a type of glial cell that have endothelial characteristics (Carr et al., 2019; Yin et al., 2022); they possess regenerative and repair potential through proliferation and migration (Piña-Oviedo and Ortiz-Hidalgo, 2008; Kucenas, 2015). After nerve injury, PCs may be among the first cells to migrate to the injured site, where they help macrophages to clear cellular debris; they may also participate in guiding the migration and regeneration of Schwann cells and nerve fibers (Lewis and Kucenas, 2014; Chen et al., 2021). Consequently, in the early stages of peripheral nerve defects, effective treatment requires the promotion of proliferation and migration in PCs and the facilitation of blood–nerve barrier formation.

The orthotopic injection of cells or bioactive substances to optimize the microenvironment for nerve regeneration has created new opportunities for treating peripheral nerve defects (Xu et al., 2024; Yang et al., 2024). Adult stem cell transplantation has been extensively researched because of its diverse sources, multidirectional differentiation potential, and robust regenerative capacity (Yi et al., 2020; Zhang et al., 2021). Previous stem cell transplantation therapies have primarily concentrated on mesoderm-derived adult stem cells, such as bone marrow mesenchymal stem cells, which do not share homology with peripheral nerves that develop from the ectoderm (Li et al., 2021; Yao et al., 2025). Moreover, ectoderm-derived adult stem cells, such as olfactory stem cells, are limited in availability and relatively challenging to obtain. By contrast, hair follicle NCSCs (hfNCSCs) represent a type of ectoderm-derived adult stem cells that share homology with peripheral nerves and are both abundant and easily accessible (Hejazian et al., 2022; Peterson and Nair, 2022). Previous studies have confirmed that hfNCSCs can facilitate facial nerve regeneration and functional recovery in rat models (Lv et al., 2024; Zhang et al., 2024a). Nonetheless, the application of stem cell transplantation carries inherent risks, particularly regarding immune rejection.

Small extracellular vesicles (sEVs), which are secreted by cells, play a crucial role in paracrine signaling (Cunha E Rocha et al., 2024; Welsh et al., 2024). These nanoscale vesicles have diameters ranging from 30 to 200 nm, and are capable of transporting a variety of bioactive substances from cells, thereby mitigating several risks associated with stem cell transplantation, including immune rejection and tumor formation. Consequently, sEVs secreted by hfNCSCs (hfNCSC-sEVs) have garnered much attention. Exosomes secreted by hfNCSCs reportedly promote tissue repair in facial nerve defects (Pan et al., 2023); however, the specific underlying mechanisms and effects on PCs remain unclear.

Our preliminary experimental results indicated that hfNCSCs can enhance the proliferation and migration of cultured PCs *in vitro* through paracrine signaling (Yu et al., 2021). Considering the characteristics and potential applications of hfNCSC-sEVs, the present study aimed to further explore these mechanisms. MicroRNAs (miRNAs), which are abundant in hfNCSC-sEVs, regulate protein expression by inhibiting mRNA translation or inducing the degradation of target mRNA (Shi et al., 2022; Ma et al., 2023). By performing mRNA and miRNA sequencing, as well as bioinformatic analysis and experimental validation, on regenerating tissue following peripheral nerve defects and hfNCSC-sEV treatment, we aimed to elucidate the effects and mechanisms of hfNCSC-sEVs on PCs, thereby providing a novel strategy for the clinical treatment of peripheral nerve defects.

## Methods

### Isolation and culture of hair follicle neural crest stem cells and perineurial cells

Sixty-five (57 for *in vivo* experiments and eight for primary cell culture experiments) healthy, naïve male Sprague–Dawley rats (approximately 4 or 8 weeks old, specific pathogen-free grade) were purchased from the Experimental Animal Center of the Naval Medical University (license No. SYXK (Hu) 2022-0011) and housed at a maximum of three rats per cage. The rodents had free access to water and food and were allowed to acclimate for 7 days prior to the experiment. The animals were maintained at approximately 22°C and 55% humidity, with a 12-hour light/dark cycle. Experimental procedures received ethical approval from the Committee on Ethics of Medicine at Navy Medical University on February 8, 2022, and were conducted in accordance with the National Institutes of Health Guide for the Care and Use of Laboratory Animals (8^th^ ed., National Research Council, 2011).

Male rats weighing approximately 80 g were anesthetized in an induction chamber pre-filled with 4% isoflurane gas (RWD, Shenzhen, China) and were subsequently euthanized via cervical dislocation. Hair follicle bulges and perineuria were dissected and isolated on ice. The samples were then placed in a 12-well plate and incubated at 37°C with 5% CO_2_ until they were ready for subculturing. Subsequently, the primary cells were digested with 0.25% trypsin-ethylenediaminetetraacetic acid (Cat# 25200, Gibco, Grand Island, NY, USA) for 10 seconds to eliminate Schwann cells, followed by the digestion of the remaining adherent cells. The cells were then seeded and allowed to adhere for 30 minutes to allow fibroblast attachment. Next, the cell suspension was transferred to a new culture dish to obtain PCs. Using this method, the PCs were purified and subcultured to the third generation. For further characterization, these cells, along with hfNCSCs subcultured to the third generation, were subjected to immunofluorescence staining to identify specific cell markers.

### Extraction, identification, and uptake of small extracellular vesicles derived from hair follicle neural crest stem cells

Medium containing hfNCSC-sEVs from the third generation was collected. The hfNCSC-sEVs were subsequently isolated and purified using an exosome isolation and purification kit (Cat# UR52121, Umibio, Shanghai, China) following the manufacturer’s instructions. Specific surface markers of sEVs were quantified using western blot analysis. The diameter distribution of hfNCSC-sEVs was analyzed using a nanoparticle tracking analyzer (NS300, Malvern Panalytical, Malvern, UK). The morphology of hfNCSC-sEVs was observed using transmission electron microscopy (TEM; 1230, JEOL, Tokyo, Japan). The following antibodies were used for western blot analysis: rabbit polyclonal anti-cluster of differentiation (CD)9 antibody (1:1000, Cat# SAB4503606, Sigma, St. Louis, MO, USA), mouse monoclonal anti-CD81 antibody (1:1000, Cat# ab109201, Abcam, Cambridge, UK), mouse monoclonal anti-tumor susceptibility gene 101 protein (TSG101) antibody (1:1000, Cat# MA1-23296, Invitrogen, Carlsbad, CA, USA), and rabbit polyclonal anti-calnexin antibody (1:1000, Cat# ab22595, Abcam). The hfNCSC-sEVs were labeled using the exosome red fluorescent labeling dye PKH26 (Cat# UR52302, Umibio), and the uptake of labeled hfNCSC-sEVs by PCs was assessed following a 36-hour incubation period.

### Cell proliferation assay

PCs were inoculated into 96-well plates at a density of 2000 cells/mL and were cultured for 24 hours. The hfNCSC-sEVs were then added to each well at the corresponding concentrations. Absorbance values at 450 nm were measured for each group of cells at 3, 5, and 7 days post-inoculation using a Cell Counting Kit-8 (CCK-8) assay kit (Cat# CA1210, Solarbio, Beijing, China) following the manufacturer’s instructions. Nuclei of proliferating PCs were stained using a Click-iT EdU-488 cell proliferation assay kit (Cat# G1601, Servicebio, Wuhan, China) according to the manufacturer’s instructions. The cell proliferation rate was calculated by determining the ratio of 5-ethynyl-2′-deoxyuridine-positive nuclei to the total number of nuclei.

### Cell migration assay

For the Transwell assay, a suspension of PCs was inoculated into the upper chamber of Transwell inserts (24-well plate, 8 μm) at a density of 1.5 × 10^4^ cells per well in a volume of 200 μL. Subsequently, 800 μL of Dulbecco’s Modified Eagle Medium/F12 medium (Cat# 10-092-CVRC, Corning, New York, NY, USA) containing 5% fetal bovine serum (Cat# 35-081-CV, Corning), 1% penicillin/streptomycin (Cat# 15140122, Gibco), and the corresponding concentration of hfNCSC-sEVs was added to each lower chamber. After 6, 18, and 24 hours, migrated cells were fixed before being stained with crystal violet solution (Cat# 60506ES60, YEASEN, Shanghai, China). The stained inserts were then examined under an optical microscope (CKKX53, Olympus, Tokyo, Japan) and the numbers of migrating cells were recorded.

For the wound healing assay, PCs were resuspended in specific culture medium for each group and inoculated onto a six-well plate at a density of 5 × 10^5^ cells per well. Once the cells reached 90% confluence, a wound was manually created in the monolayer using a sterile pipette tip. Images were captured at 0, 12, and 24 hours using an optical microscope, and the scratch area was analyzed using ImageJ software (version 1.8.0; National Institutes of Health, Bethesda, MD, USA). The cell migration rate was calculated as the ratio of the change in scratch area to the initial scratch area.

### Cell tubule formation assay

Using pre-cooled pipette tips, Matrigel was uniformly distributed into the wells of a 96-well plate, which was kept on ice. The PCs were resuspended in the culture medium specific to each group and then added to each well as a cell suspension (density: 2 × 10^4^ cells per well). The formation of tubular structures was observed under an optical microscope at 6, 12, 18, and 24 hours after cell inoculation, and a quantitative analysis was conducted to measure the numbers of junctions and total lengths of tubes using the Angiogenesis Analyzer plugin in ImageJ software (Carpentier et al., 2020).

### Transmembrane electrical resistance measurements and cell monolayer permeability assays

PCs were resuspended in the specific culture medium for each group. A total of 2.5 × 10^4^ cells in 200 μL of suspension per well were inoculated into the upper chamber of Transwell inserts (24-well plate, 0.4 μm). Next, 800 μL of Dulbecco’s Modified Eagle Medium/F12 medium (Cat# 10-092-CVRC, Corning) containing 5% fetal bovine serum (Cat# 35-081-CV, Corning) and 1% penicillin/streptomycin (Cat# 15140122, Gibco) was added to the lower chamber. The transmembrane resistance value of the upper chamber was measured using a resistance meter (MERS00002, Millicell, Burlington, MA, USA) every 24 hours. On day 7 after cell inoculation, 1 mg/mL of fluorescein isothiocyanate-dextran (Cat# ST2935, Sigma) was added to the top of each chamber. After a 30-minute incubation period, 100 μL of solution from the lower chamber of each well was collected, and its fluorescence was measured using a multifunctional microplate reader (Infinite 200PRO, Tecan, Männedorf, Switzerland). Fluorescence was quantified using Tecan i-control software (version 1.9; TECAN, Männedorf, Switzerland) with an excitation wavelength set at 485 nm and an emission wavelength set at 527 nm.

### Preparation of small extracellular vesicles derived from hair follicle neural crest stem cells loaded with miR-21-5p inhibitor

Using the ExoLoad kit (Cat# ELSR-06, ECHO-biotech, Beijing, China), miR-21-5p inhibitor, hfNCSC-sEVs, and EV-Transit Peptide were mixed sequentially in the specified proportions and incubated following the manufacturer’s instructions. Next, the samples were transferred to an ultrafiltration tube to eliminate free oligonucleic acids, and hfNCSC-sEVs loaded with miR-21-5p inhibitor were obtained.

### Animal experiments

Male rats weighing approximately 230 g had anesthesia induced with 4% isoflurane and maintained with 2% isoflurane using facemask anesthesia. The right sciatic nerve was transected approximately 1 cm from the sciatic notch, resulting in a 5-mm defect with neat transection ends. The nerve ends were then sutured into a silicon tube (7 mm length, 1.5 mm internal diameter, and 2 mm external diameter) to a depth of 1 mm. In the hfNCSC-sEVs group, 10 µL of hfNCSC-sEVs at a concentration of 1.5 × 10^10^ particles/mL was administered, whereas the phosphate-buffered saline (PBS) group received approximately 10 µL of PBS. In the Autograft group, a 5-mm segment of the sciatic nerve was excised from the same location, reversed, and sutured back into place. In the Sham group, the sciatic nerve remained untreated. Additionally, in the hfNCSC-sEVs + miR-21-5p inhibitor group, 10 µL of hfNCSC-sEVs loaded with miR-21-5p inhibitor was introduced into the silicon tube.

### Western blotting

Proteins were isolated from PCs, hfNCSCs, or regenerated tissue using radioimmunoprecipitation assay lysis buffer (Cat# P0013B, Beyotime, Shanghai, China), and their concentrations were quantified using the bicinchoninic acid protein assay kit (Cat# P0012S, Beyotime). The samples were denatured by heating before being separated using sodium dodecyl sulfate polyacrylamide gel electrophoresis (EpiZyme, Shanghai, China). Following transfer to a polyvinylidene fluoride membrane (EpiZyme), the membrane was blocked using the blocking buffer (Cat# G2052, Servicebio) at room temperature for 1 hour. It was then incubated with primary antibody at 4°C overnight, followed by washing and incubation with secondary antibody at room temperature (25°C) for 2 hours. Finally, the protein bands were visualized for detection, and quantitative analysis was performed using ImageJ software. The following primary antibodies were used: rabbit monoclonal anti-proliferating cell nuclear antigen (PCNA) antibody (1:2000, Cat# 60097-1-Ig, Proteintech, Wuhan, China), rabbit monoclonal anti-vimentin antibody (1:1000, Cat# 5741, Cell Signaling Technology, Danvers, MA, USA), rabbit polyclonal anti-claudin-1 antibody (1:1000, Cat# 13050-1-AP, Proteintech), rabbit polyclonal anti-zonula occludens 1 (ZO1) antibody (1:10 000, Cat# 21773-1-AP, Proteintech), rabbit polyclonal anti-mothers against decapentaplegic homolog (SMAD)7 antibody (1:500, Cat# WL02975, Wanleibio, Shenyang, China), rabbit polyclonal anti-SMAD2/3 antibody (1:1000, Cat# WL01520, Wanleibio), rabbit polyclonal anti-p-SMAD2/3 antibody (1:500, Cat# WL02305, Wanleibio), rabbit recombinant anti-hyaluronan synthase 2 (HAS2) antibody (1:500, Cat# DF13702, Affinity, Cincinnati, OH, USA), rabbit monoclonal anti-β-actin antibody (1:1000, Cat# 4970, Cell Signaling Technology), and mouse monoclonal anti-β-tubulin antibody (1:5000, Cat# M20005, Abmart, Shanghai, China). Horseradish peroxidase-conjugated goat anti-mouse immunoglobulin (Ig)G (1:5000, Cat# SA00001-1, Proteintech) and horseradish peroxidase-conjugated goat anti-rabbit IgG (1:5000, Cat# SA00001-2, Proteintech) were used as secondary antibodies.

### Hematoxylin-eosin staining and Masson’s trichrome staining

Rats from each group were perfused with 4% paraformaldehyde, and the regenerated tissue and bilateral gastrocnemius muscles were harvested. The bilateral gastrocnemius muscles were photographed and weighed to calculate the wet weight ratio of the operated muscle to its non-operated counterpart. Subsequently, tissue was fixed in the appropriate fixative, dehydrated through a series of ethanol solutions, cleared with xylene, embedded in paraffin, and sectioned. Staining was then conducted using a hematoxylin-eosin stain kit (Cat# G1120, Solarbio) and modified Masson’s trichrome stain kit (Cat# G1346, Solarbio) according to the provided instructions. Images were captured using an optical microscope, and the cross-sectional areas of muscle fibers were measured using ImageJ software.

### Immunofluorescence staining

PCs and hfNCSCs cultured *in vitro*, as well as sections of regenerated nerve tissue, were fixed with 4% paraformaldehyde at room temperature. The samples then underwent membrane permeabilization and blocking. Next, they were incubated overnight with primary antibody at 4°C, followed by incubation with secondary antibody for 2 hours at room temperature. After counterstaining with 4′,6-diamidino-2-phenylindole (DAPI; Cat# C1006, Beyotime), images were recorded using a fluorescence microscope (CKKX53, Olympus). The integrated optical density and the number and diameter of myelinated fibers were calculated using ImageJ software. The following primary antibodies were used: rabbit polyclonal anti-p75 neurotrophin receptor (p75) antibody (1:100, Cat# 55014-1-AP, Proteintech), mouse monoclonal anti-nestin antibody (1:100, Cat# MAB353, Sigma), rabbit polyclonal anti-claudin-1 antibody (1:250, Cat# 13050-1-AP, Proteintech), rabbit polyclonal anti-ZO1 antibody (1:200, Cat# 21773-1-AP, Proteintech), rabbit polyclonal anti-glucose transporter 1 (GLUT1) antibody (1:500, Cat# 21829-1-AP, Proteintech), rabbit monoclonal anti-S100 antibody (1:800, Cat# MAB353, Abcam), mouse monoclonal anti-neurofilament 200 (NF200) antibody (1:800, Cat# N5389, Sigma), rabbit polyclonal anti-myelin basic protein (MBP) antibody (1:400, Cat# 10458-1-AP, Proteintech), mouse monoclonal anti-β-tubulin antibody (1:1000, Cat# M20005, Abmart), and rabbit polyclonal anti-HAS2 antibody (1:200, Cat# DF13702, Affinity). Alexa Fluor 488 goat anti-mouse IgG (1:800, Cat# 4408, Cell Signaling Technology), Alexa Fluor 594 goat anti-rabbit IgG (1:800, Cat# 8889, Cell Signaling Technology), Alexa Fluor 488 goat anti-rabbit IgG (1:800, Cat# 4412, Cell Signaling Technology), and Alexa Fluor 594 goat anti-mouse IgG (1:800, Cat# 8890, Cell Signaling Technology) were used as secondary antibodies.

### Transmission electron microscopy

The central portion of regenerated tissue was dissected into small fragments and fixed in TEM fixative (Cat# G1102, Servicebio) for 2 hours at 4°C. The samples were then dehydrated in ethanol with an increasing concentration gradient, embedded in epoxy resin, and sectioned into ultra-thin slices (80 nm). The samples were stained in the dark for 8 minutes using a 2% uranyl acetate (Cat# 02624-AB, SPI, West Chester, PA, USA) saturated ethanol solution. After washing, the samples were further stained with a 2.6% lead citrate(Cat# 02690-AB, SPI) solution under CO₂-free conditions for 8 minutes. Finally, the copper grids carrying the ultrathin sections were dried overnight at 25°C. The axonal structure was examined using a TEM (Tecnai G2 Spirit, FEI, Hillsboro, OR, USA). Myelin ultrastructure was assessed, and the G-ratio, defined as the ratio of the axon diameter to the total fiber diameter (i.e., axon plus myelin), was quantitatively analyzed using ImageJ software.

### Walking track analysis

At 10 weeks post-surgery, the rats in each group underwent walking track analysis to evaluate their motor function. To capture their paw prints, the hind paws of the rats were dipped in ink before they were allowed to walk along a path. The paw prints were subsequently analyzed to calculate the sciatic functional index (SFI) using the formula: SFI = −38.3 × (EPL − NPL) / NPL + 109.5 × (ETS − NTS) / NTS + 13.3 × (EIT − NIT) / NIT − 8.8, where PL denotes the span from the third toe to the heel, TS refers to the distance between the first and fifth toes, and IT indicates the separation between the second and fourth toes. The operands E and N represent the limbs that underwent surgery and those that did not, respectively.

### Electrophysiology

At 10 weeks post-surgery, rats in each group were anesthetized with isoflurane, and the injured sciatic nerve was exposed. Compound muscle action potentials (CMAP) were recorded using an electromyography evoked potential instrument (Keypoint, Alpine BioMed ApS, Skovlunde, Denmark). The recording electrode was inserted into the medial belly of the ipsilateral gastrocnemius muscle, and the ground electrode was connected to the tail tip. A pair of bipolar hook electrodes was used to contact the distal and proximal sites of the sciatic nerve injury, and an electrical stimulus of 10 mA was applied to elicit the CMAP response. The mean amplitude and latency of the CMAP were recorded for each rat. Motor nerve conduction velocity was determined by dividing the distance between the proximal and distal stimulation electrodes by the difference in latency. Sensory nerve conduction velocity was calculated as the ratio of the distance between the stimulation and recording electrodes to their corresponding latencies.

### mRNA sequencing and data analysis

Total RNA was extracted from regenerated tissue 7 days post-surgery in both the PBS and hfNCSC-sEVs groups using TRIzol reagent (Cat# R0016, Beyotime). The quality and quantity of the RNA were evaluated using a spectrophotometer (NanoDrop 2000, Thermo Fisher Scientific, Waltham, MA, USA). RNA integrity was examined using the Agilent 2100 Bioanalyzer (Agilent Technologies, Santa Clara, CA, USA). Following the provided instructions, RNA libraries were constructed using a TruSeq stranded mRNA LT sample prep kit (Illumina, San Diego, CA, USA). The sequencing of the transcriptome and its subsequent analysis were conducted by OE Biotech Co., Ltd. (Shanghai, China). Among all differentially expressed genes, those associated with endothelial cell proliferation and migration were selected for volcano plot and clustering heatmap visualization. Subsequently, Gene Ontology (GO) Analysis (http://www.geneontology.org) and Kyoto Encyclopedia of Genes and Genomes (KEGG) Analysis (http://www.genome.ad.jp/kegg/) were conducted for these genes, and those with the highest correlation to both cell proliferation and migration were further identified.

### Small RNA sequencing

Total RNA was extracted from hfNCSC-sEVs using TRIzol reagent (Cat# 15596018CN, Thermo Fisher Scientific). Subsequently, small RNA sequencing was performed on the Illumina HiSeq sequencing platform, and a single-end library was constructed for SE50 sequencing. The resulting sequencing data underwent quality control and subsequent bioinformatic analyses to evaluate the high-quality data. The miRNA sequencing and analysis of hfNCSC-sEVs were performed by Umibio.

### Quantitative reverse transcription-polymerase chain reaction

Total RNA was extracted from PCs in each group using TRIzol reagent (Thermo Fisher Scientific) according to the manufacturer’s instructions. The miRNA expression levels were analyzed using a Hairpin-it^TM^ miRNA quantitative reverse transcription-polymerase chain reaction (qRT-PCR) quantitation kit (Cat# E01006, GenePharma, Suzhou, China) in accordance with the instructions. U6 served as the reference gene. The cycle threshold (CT value) was obtained using Bio-Rad CFX Maestro software (version 4.0.2325.0418, Bio-Rad, Hercules, CA, USA). The 2^–ΔΔCT^ method was used to calculate and analyze miRNA expression levels. All primer sequences are listed in **Table 1**, and were provided by Sangon Biotech Co., Ltd. (Shanghai, China).

**Table 1:** Primer sequences used for quantitative reverse transcription-polymerase chain reaction

Primer	Primer sequence (5'–3')	Product size (bp)
rno-miR-21-5p-F	ACA CTC CAG CTG GGT AGC TTA TCA GAC TGA	66
rno-miR-21-5p-R	TGG TGT CGT GGA GTC G	
U6-F	CTC GCT TCG GCA GCA CA	94
U6-R	AAC GCT TCA CGA ATT TGC GT	

### Cell treatment and transfection

For cell treatment, transforming growth factor (TGF)-β (Cat# HY-P70648, MedChemExpress, Monmouth Junction, NJ, USA) was administered at a concentration of 5 ng/mL. miR-21-5p mimics or si-*Has2*, provided by Sangon Biotech Co., Ltd., were transfected using Lipofectamine RNAiMAX reagent (Cat# L13778150, Invitrogen) according to the instructions. Non-specific small interfering RNA (siRNA), provided by Sangon Biotech Co., Ltd., served as a control. The PCs were inoculated into six-well plates and incubated with miR-21-5p mimics, si-Has2, or non-specific siRNA for 24–48 hours to achieve stable transfections.

### Dual-luciferase reporter assay

Luciferase vectors containing either the wild-type (WT) or mutant 3ʹ-untranslated region (UTR) of *Smad7*, as well as miR-21-5p or a negative control, were constructed by OBIO Co., Ltd. (Shanghai, China). Subsequently, 293T cells (Cat# SCSP-502, Cell Bank of Chinese Academy of Sciences, Shanghai, China) were co-transfected with these luciferase vectors using Lipofectamine 2000 (Cat# L11668030, Invitrogen). After 48 hours, the dual-luciferase reporter gene assay system (Cat# E1910, Promega, Madison, WI, USA) was used following the manufacturer’s instructions. Relative luciferase activity (Renilla luciferase/firefly luciferase) was calculated to assess the regulatory effect of miR-21-5p on its putative target gene, *Smad7*.

### Statistical analyses

Statistical analyses were performed using GraphPad Prism (version 10.1.2, GraphPad Software, Boston, MA, USA). Results are presented as the mean ± standard error of the mean. Comparisons between two groups were conducted using Student’s *t*-test, whereas comparisons among multiple groups were performed using one-way analysis of variance followed by Tukey’s *post hoc* test for pairwise comparisons. Significance was determined at a *P*-value threshold of 0.05.

## Results

### Small extracellular vesicles derived from hair follicle neural crest stem cells are taken up by perineurial cells *in vitro* and enhance their proliferation and migration

**Figure 1:**
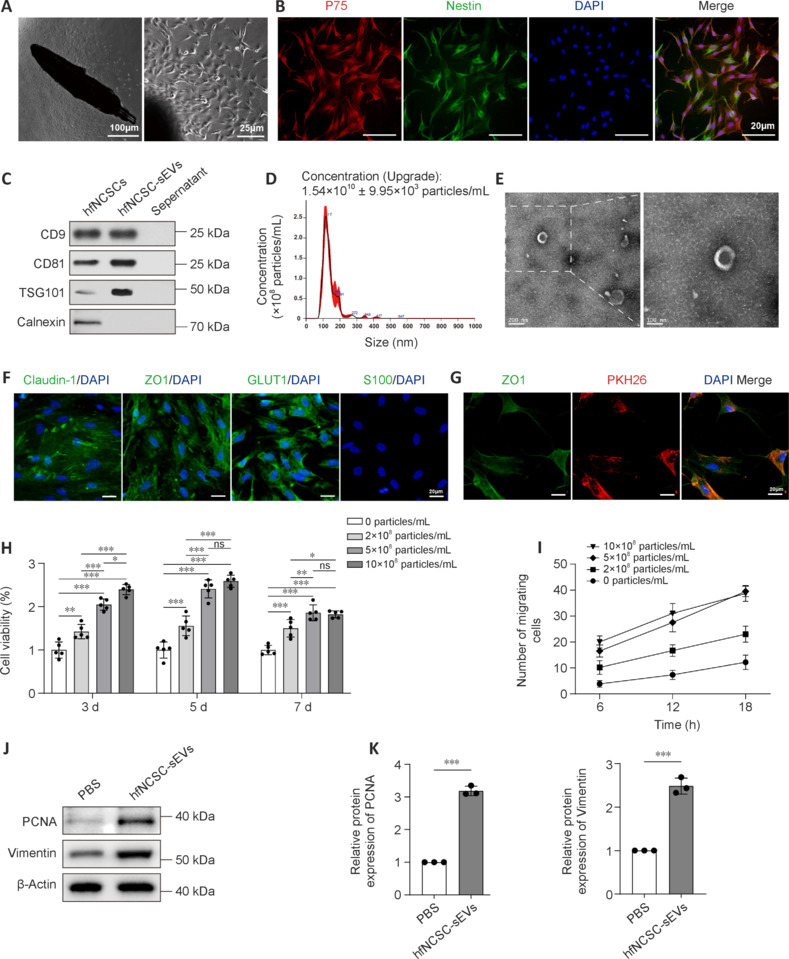
hfNCSC-sEVs are taken up by PCs *in vitro* and enhance their proliferation and migration. (A) Primary cultures of hfNCSCs were established from male Sprague–Dawley rats. (B) Immunofluorescence staining of the neural crest cell marker p75 (red) and the stem cell marker nestin (green) in hfNCSCs, with 4′,6-diamidino-2-phenylindole (DAPI) staining indicating the nuclei. (C) Western blot analysis demonstrated the presence of surface markers (cluster of differentiation [CD]9, CD81, and tumor susceptibility gene 101 protein [TSG101]) and the absence of an endoplasmic reticulum marker (calnexin) in hfNCSC-sEVs. (D) Nanoparticle tracking analysis was used to quantify the concentration and size distribution of hfNCSC-sEVs. (E) Transmission electron microscopy was used to visualize the characteristic morphology of hfNCSC-sEVs. (F) Immunofluorescence staining indicated that the third-generation PCs cultured *in vitro* were positive for claudin-1, zonula occludens 1 (ZO1), and glucose transporter 1 (GLUT1) but negative for S100, with DAPI staining marking the nuclei. (G) The internalization of PKH26-labeled hfNCSC-sEVs (red) by ZO1-positive PCs (green) was visualized using immunofluorescence staining, with DAPI staining to mark the nuclei. (H) The Cell Counting Kit-8 assay was used to evaluate the cell viability of PCs across concentrations of 0, 2 × 10^8^, 5 × 10^8^, and 10 × 10^8^ particles/mL hfNCSC-sEVs at 3, 5, and 7 days of *in vitro* culture (*n* = 5 per group). (I) The Transwell assay was used to quantify the number of migrating PCs at 6, 12, and 18 hours post-treatment with the aforementioned concentrations of hfNCSC-sEVs, in *in vitro* culture (*n* = 6 per group). (J) Western blot and (K) statistical analyses revealed the relative protein expression levels of proliferating cell nuclear antigen (PCNA) and vimentin in PCs from the phosphate-buffered saline (PBS) and hfNCSC-sEVs groups on day 5 of *in vitro* culture (normalized to β-actin, *n* = 3 per group). Data are expressed as the mean ± SEM. *P < 0.05, **P < 0.01, ***P < 0.001 (one-way analysis of variance and Tukey’s multiple comparison test for H and I; Student’s *t*-test for K). The data were from at least three separate and independent studies. CCK-8: Cell counting kit-8; GLUT1: glucose transporter 1; hfNCSCs: hair follicle neural crest stem cells; ns: not significant; PCNA: proliferating cell nuclear antigen; PCs: perineurial cells; sEVs: small extracellular vesicles; ZO1: zonula occludens 1.

To obtain hfNCSCs, rat hair follicle bulges were cultured using the tissue explant method. By day 4 post-explantation, hfNCSCs started to emerge from the tissue, and primary hfNCSCs were successfully isolated by day 7 (**Figure 1A**). Immunofluorescence staining demonstrated the strong expression of the neural crest cell marker p75 and the stem cell marker nestin in hfNCSCs following four purification passages (**Figure 1B**). Supernatant from the hfNCSC culture medium was collected, and hfNCSC-sEVs were subsequently isolated and purified. Western blot analysis confirmed the presence of the surface markers CD9, CD81, and TSG101 in hfNCSC-sEVs; however, the endoplasmic reticulum marker calnexin was absent (**Figure 1C**). Nanoparticle tracking analysis revealed that the average particle size of hfNCSC-sEVs was approximately 117 nm, with a concentration of about 1.54 × 10^10^ particles/mL (**Figure 1D**). TEM demonstrated that hfNCSC-sEVs exhibited a round or oval cup-like vesicle structure (**Figure 1E**). Together, these results suggest that hfNCSC-sEVs were obtained at an appropriate concentration. Furthermore, immunofluorescence staining indicated that the specific markers claudin-1, ZO1, and GLUT1 were highly expressed in cultured and purified PCs, whereas the Schwann cell marker S100 was not detected (**Figure 1F**). Additionally, immunofluorescence staining revealed the presence of PKH26-labeled hfNCSC-sEVs within PCs (**Figure 1G**), indicating that PCs can take up hfNCSC-sEVs *in vitro*.

Initially, the effects of hfNCSC-sEVs on the proliferative and migratory capabilities of PCs were evaluated *in vitro*. The CCK-8 assay revealed that, compared with the control group (0 particles/mL), the cell viability of PCs cultured with varying concentrations of hfNCSC-sEVs (2 × 10^8^, 5 × 10^8^, and 10 × 10^8^ particles/mL) significantly increased on days 3, 5, and 7. The most notable increase was observed on day 5, with no significant difference identified between the groups treated with 5 × 10^8^ and 10 × 10^8^ particles/mL (**Figure 1H**). Similarly, results from the Transwell assay indicated that different concentrations of hfNCSC-sEVs substantially increased the numbers of migrating PCs. Notably, the groups treated with 5 × 10^8^ particles/mL and 10 × 10^8^ particles/mL exhibited a significant increase compared with the 2 × 10^8^ particles/mL group, and no significant difference was observed between the two higher concentration groups (**Figure 1I**). Consequently, a concentration of 5 × 10^8^ particles/mL was used for the experimental application of hfNCSC-sEVs. Western blot analysis demonstrated that on day 5 of *in vitro* culture, PCNA expression levels were significantly higher in the hfNCSC-sEVs group than in the PBS group (*P* < 0.001). Additionally, vimentin expression levels, which positively correlate with cell migration ability, were also significantly higher (*P* = 0.001; **Figure 1J** and **K**). Collectively, these findings suggest that hfNCSC-sEVs effectively enhance the proliferation and migration of PCs *in vitro*.

### Small extracellular vesicles derived from hair follicle neural crest stem cells enhance tube formation and barrier function and upregulate tight junction protein expression in perineurial cells

**Figure 2:**
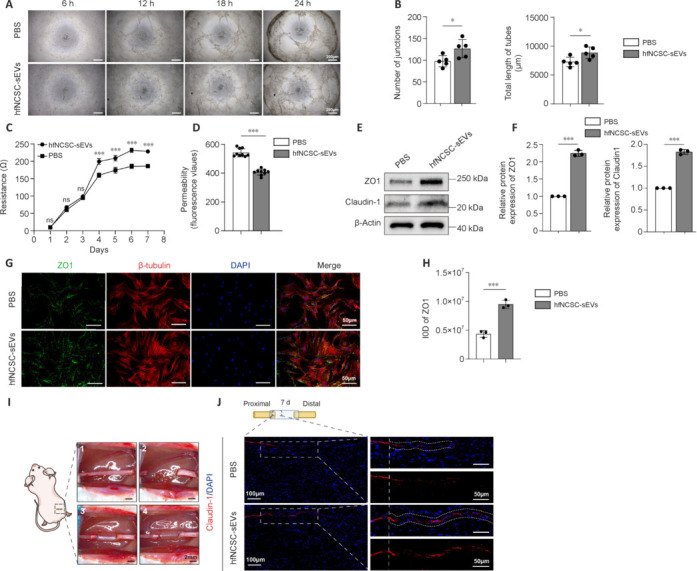
hfNCSC-sEVs enhance tube formation and barrier function in PCs and promote tight junction protein expression. (A) Optical micrographs of the tube formation assay and (B) statistical analyses demonstrated the number of junctions and total length of tubes in PCs in both the phosphate-buffered saline (PBS) and hfNCSC-sEVs groups (*n* = 5 per group). (C) Measurements of transmembrane resistance (*n* = 3 per group) and (D) cell monolayer permeability assays (*n* = 9 per group) indicated the barrier formation ability of PCs in both the PBS and hfNCSC-sEVs groups. (E) Western blot and (F) statistical analyses revealed the relative protein expression levels of the tight junction proteins zonula occludens 1 (ZO1) and claudin-1 in PCs from the PBS and hfNCSC-sEVs groups on day 7 of *in vitro* culture (normalized to β-actin, *n* = 3 per group). (G, H) Immunofluorescence staining (G) and statistical analyses (H) showed the integrated optical density (IOD) of ZO1 (green) and the expression of β-tubulin (red) in PCs from the PBS and hfNCSC-sEVs groups on day 7 of *in vitro* culture (*n* = 3 per group). (I) Schematic illustration of the rat sciatic nerve defect model: a 5-mm defect was surgically created in the rat sciatic nerve, which was then bridged using a silicon tube, followed by an orthotopic injection procedure. (J) Immunofluorescence staining revealed the expression of claudin-1 (red) in the proximal end of regenerated tissue in both the PBS and hfNCSC-sEVs groups on day 7 post-operation, with 4′,6-diamidino-2-phenylindole (DAPI) staining indicating the nuclei. Data are expressed as the mean ± SEM. *P < 0.05, ***P < 0.001 (Student’s *t*-test for B, C, D, F, and H). The data were from at least three separate and independent studies. hfNCSCs: Hair follicle neural crest stem cells; IOD: integrated optical density; PCs: perineurial cells; sEVs: small extracellular vesicles; ZO1: zonula occludens 1.

Given the endothelial cell-like properties of PCs, which are responsible for enveloping nerves to establish the perineurium, we investigated the capacity of PCs for tube formation. The tube formation assay revealed that lumina initiation occurred within 6 hours of inoculation and progressively enlarged over time. Notably, the number of junctions (*P* = 0.0291) and total length of tubes (*P* = 0.0233) of PCs in the hfNCSC-sEVs group were significantly greater than those in the PBS group at the 18-hour time point (**Figure 2A** and **B**). These findings suggest that hfNCSC-sEVs may potentiate the tubular formation of PCs *in vitro*. Furthermore, we assessed the establishment of tight junctions and their barrier functions during the membrane formation process of PCs. The transmembrane electrical resistance value of PCs in the hfNCSC-sEVs group was significantly higher than that in the PBS group on day 3, and this enhancement was maintained through day 7 (from day 3 to day 7: *P* < 0.001; **Figure 2C**). Higher resistance values indicate lower permeability, suggesting that the PCs exposed to hfNCSC-sEVs were more proficient at forming tight junctional barriers. On day 7 of membrane formation development, cell monolayer permeability assays revealed that fluorescence intensity in the lower chamber was significantly lower in the hfNCSC-sEVs group than in the PBS group (*P* < 0.001; **Figure 2D**). This finding indicates that PCs in the hfNCSC-sEVs group exhibit reduced permeability and a stronger barrier formation capacity.

Tight junction proteins—specifically, claudin-1 and ZO1 in PCs—are essential for establishing the blood–nerve barrier (Lux et al., 2019). Western blot analysis demonstrated that the protein expression levels of ZO1 (*P* < 0.001) and claudin-1 (*P* < 0.001) were significantly higher in the hfNCSC-sEVs group than in the PBS group on day 7 of *in vitro* culture (**Figure 2E** and **F**). Furthermore, immunofluorescence staining revealed that the integrated optical density for ZO1 was markedly higher in the hfNCSC-sEVs group than in the PBS group (*P* < 0.001; **Figure 2G** and **H**). Together, these findings suggest that hfNCSC-sEVs enhance tight junction protein expression in PCs and improve the capacity of PCs for tube formation and barrier functionality *in vitro*. Claudin-1 is specifically expressed in the PCs of peripheral nerves (Reinhold et al., 2018; Ben-Kraiem et al., 2021). On day 7 following the bridging of a 5-mm defect in the sciatic nerve of rats (**Figure 2I**), immunofluorescence staining at the proximal end of the regenerating tissue exhibited a continuous and organized expression pattern of claudin-1. Regenerated PCs at the nerve stumps gradually formed tight junctions between cells during the processes of proliferation and migration, with claudin-1 expression appearing intermittent and scattered. Compared with the PBS group, the hfNCSC-sEVs group exhibited a broader distribution of claudin-1 expression in regenerated tissue; this was accompanied by a notable increase in claudin-1 expression intensity (**Figure 2J**). These results indicate that hfNCSC-sEVs can promote claudin-1 expression in PCs *in vivo*.

### Small extracellular vesicles derived from hair follicle neural crest stem cells promote nerve tissue regeneration and repair following sciatic nerve defect

**Figure 3:**
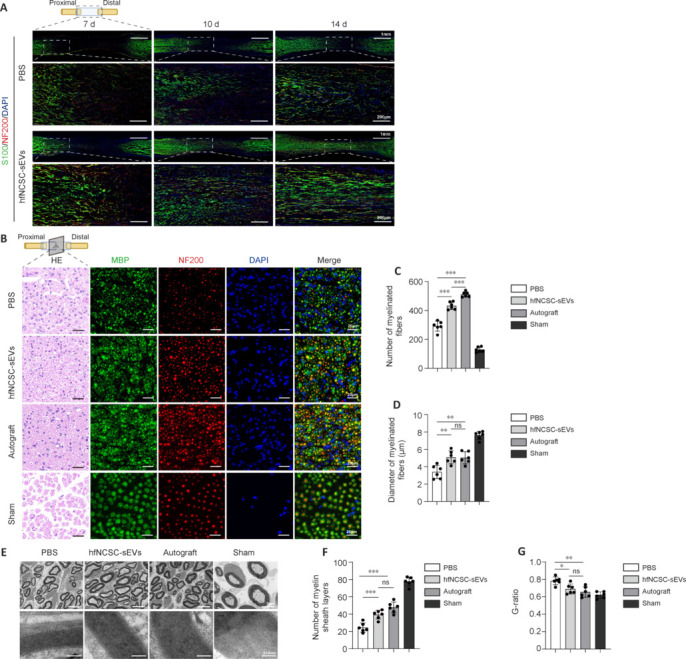
hfNCSC-sEVs facilitate nerve tissue regeneration and repair following a sciatic nerve defect. (A) Immunofluorescence staining demonstrated the expression of S100 (green), identifying Schwann cells, and neurofilament 200 (NF200; red), marking nerve fibers, within regenerated tissue in both the phosphate-buffered saline (PBS) and hfNCSC-sEVs groups at 7, 10, and 14 days post-operation, with 4′,6-diamidino-2-phenylindole (DAPI) staining indicating nuclei. (B) Hematoxylin and eosin and immunofluorescence staining revealed the expression of myelin basic protein (MBP; green), labeling the myelin sheath, and NF200 (red), highlighting nerve fibers, in a central cross-section of regenerated tissue. (C, D) Statistical analysis was used to quantify the number of myelinated nerve fibers and the diameter of these fibers across the PBS, hfNCSC-sEVs, Autograft, and Sham groups at 10 weeks post-operation (*n* = 6 per group). (E–G) Transmission electron microscopy depicted the central cross-section of regenerated tissue (E), and statistical analysis revealed the number of myelin layers (F) and G-ratio (G) among the PBS, hfNCSC-sEVs, Autograft, and Sham groups at 10 weeks post-operation (*n* = 6 per group). Data are expressed as the mean ± SEM. *P < 0.05, **P < 0.01, ***P < 0.001 (one-way analysis of variance and Tukey’s multiple comparison test for C, D, F, and G). The data were from at least three separate and independent studies. HE: Hematoxylin and eosin; hfNCSCs: hair follicle neural crest stem cells; MBP: myelin basic protein; NF200: neurofilament 200; ns: not significant; sEVs: small extracellular vesicles.

Our initial focus was to elucidate the role of hfNCSC-sEVs during the early stages of nerve repair. Immunofluorescence staining revealed that S100-positive Schwann cells migrated from both ends toward the center on days 7, 10, and 14 postoperatively. Concurrently, NF200-positive nerve fibers elongated from the proximal to the distal region. The extent of Schwann cell migration and nerve fiber regeneration in the hfNCSC-sEVs group was significantly greater than that observed in the PBS group at equivalent time intervals (**Figure 3A**).

Subsequently, we assessed the influence of hfNCSC-sEVs on the later stages of nerve repair. Excluding the Sham group, the Autograft group exhibited the highest quantity of myelinated nerve fibers encased in MBP-positive myelin sheaths, followed by the hfNCSC-sEVs group and then the PBS group, which demonstrated the lowest number (**Figure 3B–D**). TEM examinations at corresponding sites revealed no significant differences in the diameters of myelinated nerve fibers or the numbers of myelin sheath layers between the Autograft and hfNCSC-sEVs groups; however, both groups significantly surpassed the PBS group for the diameter of myelinated nerve fibers (PBS *vs.* hfNCSC-sEVs: *P* = 0.0019, PBS *vs*. Autograft: *P* = 0.0026) and for the number of myelin sheath layers (PBS *vs*. hfNCSC-sEVs: *P* < 0.001, PBS *vs.* Autograft: *P* < 0.001; **Figure 3E** and **F**). G-ratio analysis confirmed no significant difference in myelin regeneration between the hfNCSC-sEVs and Autograft groups, although the hfNCSC-sEVs group yielded notably superior results compared with the PBS group (*P* = 0.0215; **Figure 3G**). Collectively, these findings indicate that hfNCSC-sEVs effectively promote nerve fiber regeneration, Schwann cell migration, and myelin formation following sciatic nerve injury in rats.

### Small extracellular vesicles derived from hair follicle neural crest stem cells enhance nerve function recovery and reduce muscle atrophy following sciatic nerve injury

**Figure 4:**
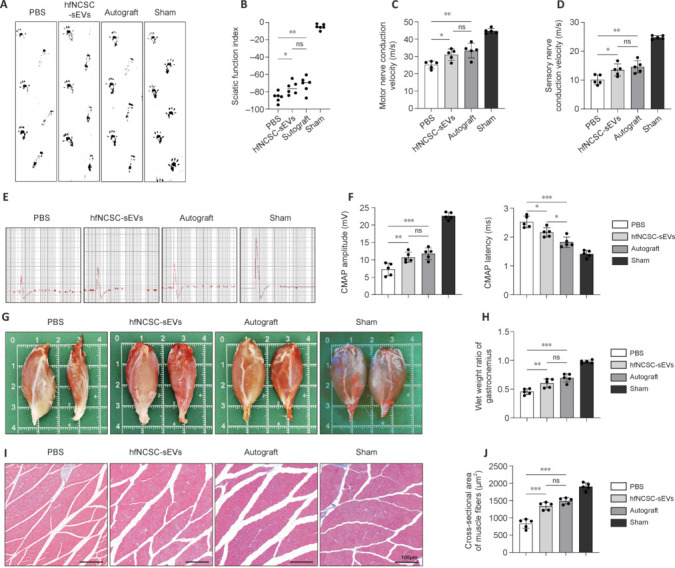
hfNCSC-sEVs enhance nerve function recovery and reduce muscle atrophy following a sciatic nerve defect. (A, B) Gait footprints (A) and the sciatic nerve function index (B) of rats in the phosphate-buffered saline (PBS), hfNCSC-sEVs, Autograft, and Sham groups were assessed at 10 weeks post-operation (*n* = 6 per group). (C, D) Motor nerve conduction velocity (C) and sensory nerve conduction velocity (D) of the regenerated tissue in the PBS, hfNCSC-sEVs, Autograft, and Sham groups at 10 weeks post-operation (*n* = 5 per group). (E, F) CMAP (E) and statistical analyses (F) were used to determine the CMAP peak amplitude and latency of the gastrocnemius on the operated side (*n* = 5 per group). (G, H) The morphology (G) of the rat gastrocnemius and the wet weight ratio (H) of the gastrocnemius were analyzed in the PBS, hfNCSC-sEVs, Autograft, and Sham groups at 10 weeks post-operation (*n* = 5 per group). (I, J) Masson staining (I) and analysis of the muscle fiber cross-sectional area (J) of the operated gastrocnemius at 10 weeks post-operation (*n* = 5 per group). Data are expressed as the mean ± SEM. *P < 0.05, **P < 0.01, ***P < 0.001 (one-way analysis of variance and Tukey’s multiple comparison test for B–D, F, H and J). The data were from at least three separate and independent studies. CMAP: Compound muscle action potential; hfNCSCs: hair follicle neural crest stem cells; ns: not significant; sEVs: small extracellular vesicles.

To evaluate motor function recovery following nerve injury, rats underwent gait analysis (**Figure 4A**), and the SFI was calculated for each group at 10 weeks post-operation. The SFI value in the Sham group was close to zero, and no significant difference was observed between the hfNCSC-sEVs and Autograft groups. However, both groups exhibited superior SFI values compared with those in the PBS group (**Figure 4B**). Electrophysiological evaluations of the injured sciatic nerve revealed no significant differences in motor or sensory nerve conduction velocity between the hfNCSC-sEVs and Autograft groups. However, both groups exhibited significantly higher values than those in the PBS group for motor nerve conduction velocity (PBS *vs.* hfNCSC-sEVs: *P* = 0.0329, PBS *vs*. autograft: *P* = 0.0027) and sensory nerve conduction velocity (PBS *vs.* hfNCSC-sEVs: *P* = 0.0305, PBS *vs*. Autograft: *P* = 0.0041; **Figure 4C** and **D**). The peak value of CMAP demonstrated a similar trend; the hfNCSC-sEVs group showed slightly lower values than those in the Autograft group. Correspondingly, the CMAP latency was significantly shorter in the Autograft group than in the hfNCSC-sEVs group, and the longest latency was observed in the PBS group (hfNCSC-sEVs *vs.* autograft: *P* = 0.0267, PBS *vs*. hfNCSC-sEVs: *P* = 0.0184; **Figure 4E** and **F**).

Sciatic nerve injury typically results in atrophy of the target muscle (the gastrocnemius), and target muscle reinnervation is a critical indicator for evaluating nerve function recovery. At 10 weeks post-operation, the gastrocnemius on the injured side exhibited significant atrophy in all groups except the Sham group (**Figure 4G**). The wet weight ratio of the gastrocnemius muscle exhibited no significant difference between the hfNCSC-sEVs and Autograft groups; however, both ratios were significantly higher than that of the PBS group (PBS *vs*. hfNCSC-sEVs: *P* = 0.0064, PBS *vs*. Autograft: *P* < 0.001; **Figure 4H**). Masson’s trichrome staining was conducted on the corresponding locations of the gastrocnemius muscle belly. The cross-sectional area of muscle fibers in both the hfNCSC-sEVs and Autograft groups was significantly larger than that in the PBS group (PBS *vs.* hfNCSC-sEVs: *P* < 0.001, PBS *vs.* Autograft: *P* < 0.001), and no significant difference was observed between the two groups (**Figure 4I** and **J**). These findings suggest that hfNCSC-sEVs facilitate nerve function restoration and alleviate target muscle atrophy following sciatic nerve injury in rats.

### miR-21-5p activates the transforming growth factor-β/mothers against decapentaplegic homolog pathway and hyaluronan synthase 2 expression

**Figure 5:**
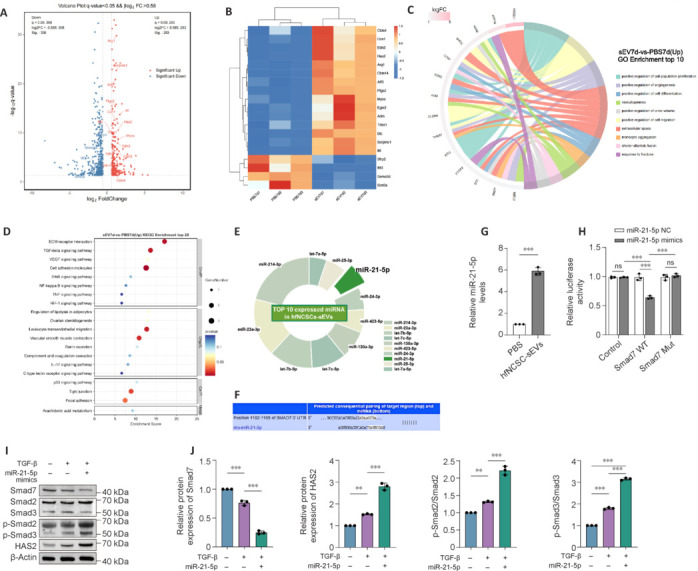
miR-21-5p activates the TGF-β/SMAD pathway by inhibiting SMAD7, resulting in upregulated HAS2 and increased PC proliferation and migration. (A, B) The volcano plot (A) and heatmap (B) illustrated the differential gene expression in regenerated tissue between the phosphate-buffered saline (PBS) and small extracellular vesicles derived from hair follicle neural crest stem cells (hfNCSC-sEVs) groups on day 7 post-operation (*n* = 3 per group). (C) The chord diagram of the Gene Ontology enrichment analysis and (D) bubble chart of the Kyoto Encyclopedia of Genes and Genomes enrichment analysis for differential genes highlighted the enriched functions and associated pathways. (E) The pie chart displayed the top 10 most abundant microRNAs (miRNAs) in hfNCSC-sEVs. (F) Schematic representation of the predicted binding sites of miR-21-5p on its target gene *Smad7*, as identified by TargetScan. (G) Quantitative reverse transcription-polymerase chain reaction results indicated the relative expression levels of miR-21-5p in PCs from the PBS and hfNCSC-sEVs groups on day 3 of *in vitro* culture (*n* = 3 per group). (H) The dual-luciferase reporter assay demonstrated the relative luciferase activity following the co-transfection of reporter constructs from the Control, wild-type (WT) *Smad7* 3′ untranslated region (UTR), and mutant *Smad7* 3′ UTR groups with either miR-21-5p overexpression plasmid or negative control vector (*n* = 3 per group). (I, J) Western blot (I) and statistical analysis (J) revealed the relative protein expression levels of SMAD7 and HAS2, as well as the phosphorylation levels of SMAD2/3 in PCs from the –/–, TGF-β/–, and TGF-β/miR-21-5p groups on day 5 of *in vitro* culture (normalized to β-actin, *n* = 3 per group). Data are expressed as the mean ± SEM. **P < 0.01, ***P < 0.001 (Student’s *t*-test for G; one-way analysis of variance and Tukey’s multiple comparison test for J, H). The data were from at least three separate and independent studies. GO: Gene Ontology; HAS2: hyaluronan synthase 2; hfNCSCs: hair follicle neural crest stem cells; KEGG: Kyoto Encyclopedia of Genes and Genomes; MUT: mutant; NC: negative control; ns: not significant; PCs: perineurial cells; sEVs: small extracellular vesicles; SMAD: mothers against decapentaplegic homolog; TGF-β: transforming growth factor-beta; WT: wild type.

To investigate the molecular mechanisms underlying the promotional effects of hfNCSC-sEVs on PC proliferation and migration during the early stages of nerve defect repair, mRNA sequencing was performed on the regenerating tissue of rats on day 7 post-surgery, comparing the PBS and hfNCSC-sEVs groups. The volcano plot revealed that 253 genes were significantly upregulated and 398 genes were significantly downregulated in the hfNCSC-sEVs group compared with the PBS group (**Figure 5A**). Among these, the differentially expressed genes associated with endothelial cell proliferation and migration were selected for cluster heatmap analysis (**Figure 5B**). Gene Ontology enrichment analysis indicated that HAS2 is implicated in both cell proliferation and migration, exhibiting the most significant difference (**Figure 5C**). Additionally, Kyoto Encyclopedia of Genes and Genomes enrichment analysis revealed the significant enrichment of biological processes related to tight junction formation and the TGF-β signaling pathway in the hfNCSC-sEVs group (**Figure 5D**). Thus, we hypothesize that hfNCSC-sEVs may upregulate HAS2 expression by activating the TGF-β pathway, thereby facilitating PC proliferation and migration.

Numerous studies have demonstrated that miRNAs are abundant in sEVs and play crucial regulatory roles in various biological functions (Tan et al., 2024; Yavuz et al., 2024; Zhang et al., 2024b). To identify the miRNAs that potentially influence PC proliferation and migration in hfNCSC-sEVs, small RNA sequencing was conducted. The top 10 known miRNAs in hfNCSC-sEVs are depicted in a pie chart (**Figure 5E**). Target genes and functional predictions, conducted using the TargetScan and miRDB databases, indicated that miR-21-5p may inhibit SMAD7 translation (**Figure 5F**), thus potentially reducing cellular sensitivity to TGF-β signaling. Consequently, the miR-21-5p present in hfNCSC-sEVs may modulate PC proliferation and migration via the TGF-β/SMAD pathway. Moreover, qRT-PCR results confirmed that the level of miR-21-5p in PCs cultured for 3 days was significantly higher in the hfNCSC-sEVs group than in the PBS group (**Figure 5G**).

A dual-luciferase reporter gene assay demonstrated that miR-21-5p overexpression reduced the activity of the *Smad7* 3′ UTR WT reporter but had no effect on the *Smad7* 3′ UTR mutant reporter (**Figure 5H**). This finding indicates that miR-21-5p can inhibit SMAD7 translation. Subsequent western blot analysis revealed that exogenous treatment with TGF-β increased the phosphorylation levels of SMAD2/3 in PCs cultured for 5 days. Furthermore, compared with the TGF-β/– group, the transfection of miR-21-5p mimics significantly downregulated SMAD7 protein expression (*P* < 0.001), further enhanced the phosphorylation levels of SMAD2 (*P* < 0.001) and SMAD3 (*P* < 0.001), and significantly upregulated HAS2 expression (*P* < 0.001; **Figure 5I** and **J**). These findings suggest that miR-21-5p may activate the TGF-β/SMAD pathway by repressing SMAD7, thereby promoting HAS2 expression in PCs.

### Hair follicle neural creast stem cells-small extreacellular vesicles promote perineurial cell proliferation and migration and upregulate tight junction protein expression by delivering miR-21-5p

**Figure 6:**
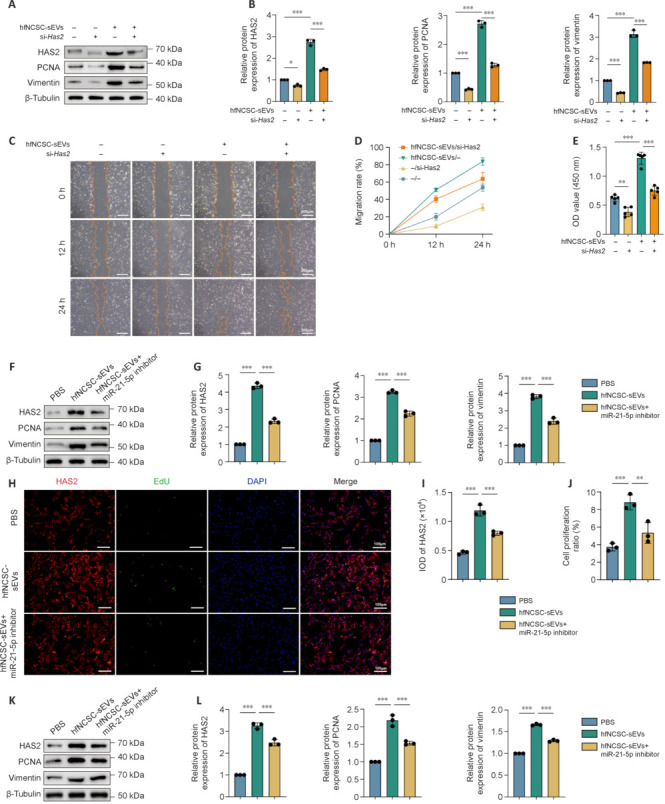
miR-21-5p in hfNCSC-sEVs augments cell proliferation and migration by enhancing HAS2 expression in PCs. (A, B) Western blot (A) and statistical analyses (B) revealed the relative protein expression levels of HAS2, proliferating cell nuclear antigen (PCNA), and vimentin in PCs across the –/–, –/si-*Has2*, hfNCSC-sEVs/–, and hfNCSC-sEVs/si-*Has2* groups on day 5 of *in vitro* culture (normalized to β-actin, *n* = 3 per group). (C, D) The wound healing assay (C) and statistical analysis (D) demonstrated the migration rates of PCs in the aforementioned groups (*n* = 3 per group). (E) The Cell Counting Kit-8 assay was used to assess cell viability in PCs across the same groups on day 5 of *in vitro* culture (*n* = 5 per group). (F, G) Western blot (F) and statistical analyses (G) indicated the relative protein expression levels of HAS2, PCNA, and vimentin in PCs treated with phosphate-buffered saline (PBS), hfNCSC-sEVs, or hfNCSC-sEVs + miR-21-5p inhibitor on day 5 of *in vitro* culture (normalized to β-actin, *n* = 3 per group). (H–J) Immunofluorescence staining visualized the expression of HAS2 (red) and 5-ethynyl-2′-deoxyuridine (EdU; green) in PCs (H), and statistical analysis revealed the integrated optical density (IOD) of zonula occludens 1 (ZO1; I) and the cell proliferation rates (J) in the PBS, hfNCSC-sEVs, and hfNCSC-sEVs + miR-21-5p inhibitor groups on day 5 of *in vitro* culture (*n* = 3 per group). (K, L) Western blot (K) and statistical analyses (L) showed the relative protein expression levels of HAS2, PCNA, and vimentin in regenerated tissue from the PBS, hfNCSC-sEVs, and hfNCSC-sEVs + miR-21-5p inhibitor groups on day 5 post-operation (normalized to β-tubulin, *n* = 3 per group). Data are expressed as the mean ± SEM. **P < 0.01, ***P < 0.001 (one-way analysis of variance and Tukey’s multiple comparison test for B, D, E, G, I, J, and L). The data were from at least three separate and independent studies. CCK-8: Cell counting kit-8; EdU: 5-ethynyl-2′-deoxyuridine; HAS2: hyaluronan synthase 2; hfNCSCs: hair follicle neural crest stem cells; IOD: integrated optical density; PCNA: proliferating cell nuclear antigen; PCs: perineurial cells; sEVs: small extracellular vesicles; ZO1: zonula occludens 1.

To examine the hypothesis that hfNCSC-sEVs enhance PC proliferation and migration through HAS2 upregulation, we used siRNA targeting *Has2* (si-*Has2*) to decrease HAS2 levels in PCs. By day 5 of *in vitro* culture, the hfNCSC-sEVs group exhibited significantly higher HAS2 (*P* < 0.001), PCNA (*P* < 0.001), and vimentin (*P* < 0.001) expression than the control group, which lacked both hfNCSC-sEVs and si-*Has2* (–/– group). Compared with the –/– and hfNCSC-sEVs/– groups, HAS2 expression was significantly downregulated in the –/si-*Has2* and hfNCSC-sEVs/si-*Has2* groups following transfection with si-*Has2* (–/– *vs*. –/si-*Has2*: *P* = 0.0134, hfNCSC-sEVs/– *vs.* hfNCSC-sEVs/si-*Has2*: *P* < 0.001). Furthermore, the PCNA (–/– *vs*. –/si-*Has2*: *P* < 0.001, hfNCSC-sEVs/– *vs*. hfNCSC-sEVs/si-*Has2*: *P* < 0.001) and vimentin (–/– *vs.* –/si-*Has2*: *P* < 0.001, hfNCSC-sEVs/– *vs.* hfNCSC-sEVs/si-*Has2*: *P* < 0.001) expression levels in these two groups were also significantly reduced (**Figure 6A** and **B**). In the wound healing and CCK-8 cell proliferation assays, suppressing HAS2 expression also markedly diminished the enhancing effects of hfNCSC-sEVs on PC proliferation and migration (**Figure 6C–E**). Collectively, these results suggest that hfNCSC-sEVs upregulate HAS2 expression in PCs, thereby facilitating their proliferation and migration.

To elucidate the role of miR-21-5p in hfNCSC-sEVs on PCs, we used an inhibitor of miR-21-5p to attenuate its activity within hfNCSC-sEVs. Western blot analysis revealed that the HAS2 (*P* < 0.001), PCNA (*P* < 0.001), and vimentin (*P* < 0.001) expression levels in PCs from the hfNCSC-sEVs group were significantly higher than those in the PBS group on day 5 of *in vitro* culture. However, this stimulatory effect was partially diminished in the hfNCSC-sEVs + miR-21-5p inhibitor group compared with the hfNCSC-sEVs group (**Figure 6F** and **G**). Immunofluorescence staining further demonstrated that the integrated optical density value of HAS2 (*P* < 0.001) and the proliferation ratio of PCs (*P* < 0.001) were markedly higher in the hfNCSC-sEVs group than in the PBS group; these effects were correspondingly reduced upon miR-21-5p inhibition (**Figure 6H–J**). Additionally, the protein expression levels of HAS2, PCNA, and vimentin in regenerated tissue decreased on day 5 post-operation when miR-21-5p was inhibited in the hfNCSC-sEVs group (**Figure 6K** and **L**).

**Figure 7:**
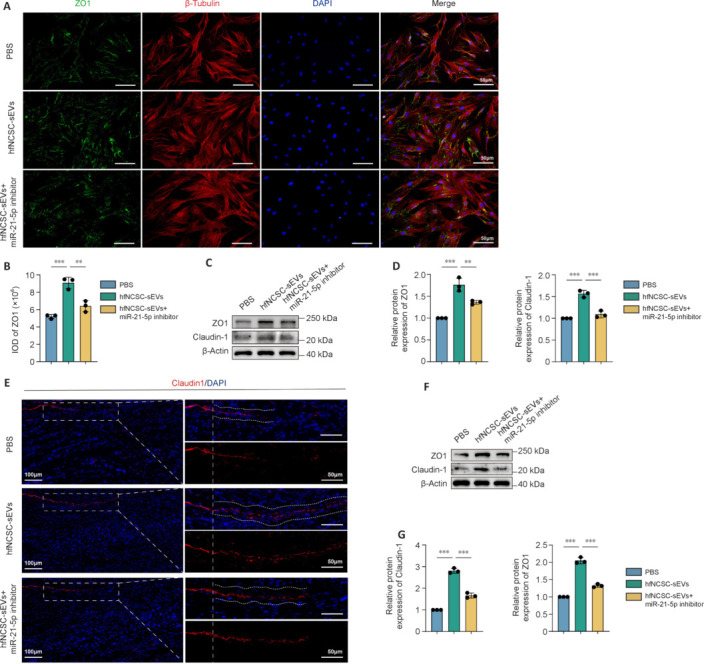
miR-21-5p in hfNCSC-sEVs enhances tight junction protein expression in PCs. (A, B) Immunofluorescence staining (A) and statistical analysis (B) demonstrated IOD of ZO1 (green) and the expression of β-tubulin (red) in PCs across the PBS, hfNCSC-sEVs, and hfNCSC-sEVs + miR-21-5p inhibitor groups on day 7 of *in vitro* culture (*n* = 3 per group). (C) Western blot and (D) statistical analyses revealed the relative protein expression levels of the tight junction proteins ZO1 and claudin-1 in PCs from the PBS, hfNCSC-sEVs, and hfNCSC-sEVs + miR-21-5p inhibitor groups on day 7 of *in vitro* culture (normalized to β-actin, *n* = 3 per group). (E) Immunofluorescence staining depicted the expression of claudin-1 (red) at the proximal end of regenerated tissue in the PBS, hfNCSC-sEVs, and hfNCSC-sEVs + miR-21-5p inhibitor groups on day 7 post-operation, with DAPI staining highlighting the nuclei. (F, G) Western blot (F) and statistical analyses (G) indicated the relative protein expression levels of ZO1 and claudin-1 in regenerated tissue across the PBS, hfNCSC-sEVs, and hfNCSC-sEVs + miR-21-5p inhibitor groups on day 7 post-operation (normalized to β-actin, *n* = 3 per group). Data are expressed as the mean ± SEM. **P < 0.01, ***P < 0.001 (one-way analysis of variance and Tukey’s multiple comparison test for B, D, and G). The data were from at least three separate and independent studies. DAPI: 4,6-Diamidino-2-phenylindole; hfNCSCs: hair follicle neural crest stem cells; IOD: integrated optical density; PBS: phosphate-buffered saline; PCs: perineurial cells; sEVs: small extracellular vesicles; ZO1: zonula occludens 1.

To further investigate the role of miR-21-5p in hfNCSC-sEVs with regard to tight junction protein formation in PCs, immunofluorescence staining and western blot analysis were used. The enhancement of tight junction protein expression by hfNCSC-sEVs was attenuated in PCs in the hfNCSC-sEVs + miR-21-5p inhibitor group on day 7 (**Figure 7A–D**). These findings were corroborated by immunofluorescence staining and western blot analysis of regenerated tissue on day 7 post-surgery (**Figure 7E–G**).

In summary, our results demonstrated that the downregulation of HAS2 in PCs treated with hfNCSC-sEVs through si-*Has2* *in vitro* significantly impacted their proliferation and migration. Notably, the application of miR-21-5p inhibitor in hfNCSC-sEVs in both PCs and regenerating tissue indicated that hfNCSC-sEVs enhanced HAS2 expression in PCs via miR-21-5p. This enhancement promoted cell proliferation and migration, which was followed by the upregulation of tight junction proteins.

## Discussion

The findings of the present study indicate that hfNCSC-sEVs facilitate the regeneration of myelin sheaths, promote the elongation of nerve fibers, and support the recovery of nerve function in the context of peripheral nerve defects. Moreover, our results suggest that hfNCSC-sEVs activate the TGF-β/SMAD signaling pathway in PCs, thereby enhancing their proliferation and migration through the delivery of miR-21-5p. In this regulatory mechanism, miR-21-5p plays a pivotal role by downregulating SMAD7 expression, which leads to the elevated phosphorylation of SMAD2/3. This cascade subsequently upregulates HAS2 expression, further augmenting the proliferation and migration capabilities of PCs. The resulting increased quantity and enhanced migratory capacity of PCs contribute to the establishment of tight junctions among cells and the formation of tubular structures, thereby strengthening the protective barrier function of the perineurium.

PCs play a crucial role in repair and regeneration processes following peripheral nerve injury, and our findings indicate that hfNCSC-sEVs significantly promote PC proliferation and migration. During neural development and growth in mice, PCs are reportedly instrumental in maintaining the organized branching and stable growth of nerves (Clark et al., 2014). Additionally, the absence of proper guidance from PCs leads to disorganized nerve growth within tissue, resulting in an increased number of branches (Chovatiya et al., 2023). Dysregulated PC growth can perturb both Schwann cell differentiation and neuromuscular junction formation. These findings imply that during peripheral nerve regeneration, the migration and guidance of PCs, as well as the integrity of the perineurium, are crucial for the accurate positioning of nerve fibers and the restoration of neural function during axonal regeneration.

Notably, the present findings indicate that activation of the TGF-β/SMAD signaling pathway substantially enhances PC proliferation and migration. Several research teams have used single-cell analysis to demonstrate that PCs exhibit mesenchymal cell characteristics after peripheral nerve injury, thus indicating their potential for regeneration and repair through proliferation and migration (Carr et al., 2019; Zhao et al., 2022, 2024). Activation of the TGF-β pathway prompts endothelial cells to initiate an endothelial–mesenchymal transition (Stenmark et al., 2016; Piera-Velazquez and Jimenez, 2019). During this process, new transcriptional programs are activated within the cells, significantly enhancing their transformation into a mesenchymal phenotype as well as their proliferation and migration (Rabelink et al., 2024). Furthermore, multiple studies have reported that TGF-β/SMAD pathway activation plays a critical role in the proliferation, migration, cellular reprogramming, and myelin remodeling of Schwann cells (Muscella et al., 2020; Hortells et al., 2021; Jessen and Mirsky, 2021). Consequently, PCs, which exhibit various functional similarities to Schwann cells, have captured our research attention. Our preliminary experimental results indicated that hfNCSCs enhance the proliferation and migration of PCs through paracrine signaling (Yu et al., 2021). The present study further confirmed that the TGF-β/SMAD signaling pathway is pivotal in the promotional effects of hfNCSC-sEVs.

In the current study, we also identified HAS2 as a critical downstream effector of the TGF-β/SMAD pathway, responsible for the proliferation and migration of PCs. HAS2 catalyzes the synthesis of hyaluronic acid, which is a crucial component of the extracellular matrix (Skandalis et al., 2020). Hyaluronic acid facilitates cell proliferation and migration by maintaining an optimal distance between cells during embryonic development, tissue repair, and regeneration. It has been reported that, as a downstream target, HAS2 is significantly upregulated in response to TGF-β stimulation (Lee et al., 2024). Similarly, numerous studies have confirmed that increased hyaluronic acid within the extracellular matrix enhances angiogenesis by facilitating cell proliferation and migration (Simińska-Stanny et al., 2024). Our experiments indicate that hfNCSC-sEVs can upregulate HAS2 expression, thereby promoting the proliferation and migration of PCs, which exhibit tube-forming properties similar to those of vascular endothelial cells.

The enhanced proliferation and migration of PCs were also associated with a subsequent upregulation of tight junction proteins. Morris et al. (2017) reported that TGF-β is pivotal in the formation of ZO1 during perineurium development in both zebrafish and mice. They also observed that blockade of the TGF-β pathway leads to a decrease in the formation of intercellular ZO1 (Morris et al., 2017; Arena et al., 2022). Nevertheless, the precise function of TGF-β in the proliferation, migration, and formation of tight junction proteins in PCs during peripheral nerve regeneration remains to be elucidated. During the endothelial–mesenchymal transition, the proliferation and migration abilities of cells increase, whereas the expression of tight junction proteins between cells decreases (Piera-Velazquez and Jimenez, 2019). Our results indicate that when PCs have migrated to their destination, their cellular characteristics begin to transition from mesenchymal to endothelial cells; this is accompanied by a significant increase in the formation of tight junctions between cells. This phenomenon may account for the observation that when PCs were cultured *in vitro* for 5 days, no significant increase in ZO1 expression was detected in the hfNCSC-sEVs group relative to the PBS group, although a marked increase was evident on day 7. Reinhold et al. (2022) reported that in cases of peripheral nerve injury, miR-21-5p diminishes claudin-1 expression in PCs via the RECK/matrix metalloproteinase 9 pathway. On the basis of our findings, we propose that this effect may be ascribed to the continuous administration of daily miR-21-5p analogs in both the *in vitro* and *in vivo* experiments, resulting in a sustained reduction in claudin-1 expression in the cells. However, in our *in vivo* and *in vitro* experiments, both hfNCSC-sEVs and miR-21-5p mimics were administered as a single dose on the initial day. The increased cell number and migratory distance observed in the early stages facilitated the subsequent assembly of tubular structures by PCs, thereby establishing a foundation for enhanced barrier function.

hfNCSC-sEVs not only exerted a multifaceted impact on PCs but also markedly enhanced Schwann cell migration, myelin sheath formation, and nerve fiber regeneration, thereby contributing to the comprehensive restoration of neural function. Extensive prior studies have validated the effectiveness of TGF-β and miR-21-5p in enhancing the repair and proliferative responses of various cell types—including nerve fibers, Schwann cells, and vascular endothelial cells—following nerve injury (Qi et al., 2024; Cong et al., 2025). Additionally, an interplay exists between PCs and Schwann cells, wherein they mutually influence each other’s proliferation and migration through communication and guidance signals, thereby supporting nerve fiber regeneration (Clark et al., 2014; Lee et al., 2020). Consequently, we speculate that the enhanced proliferative and migratory capacities of PCs may be linked to complex processes such as myelination and nerve fiber regeneration. Nonetheless, this hypothesis requires additional empirical exploration.

Our study has several limitations. First, the specific actions and mechanisms of other components within hfNCSC-sEVs (such as additional miRNAs or proteins) on PCs remain to be elucidated. Second, following peripheral nerve defects, the downstream effects of PCs forming tight junctions and the blood–nerve barrier on the internal neural environment and nerve fiber regeneration is another area that needs further exploration. We must also note that hfNCSCs exhibit developmental homology with neural tissue. In contrast to mesoderm-derived mesenchymal stem cells and other source-restricted ectodermal stem cells, sEVs derived from hfNCSCs may encapsulate additional mechanisms that facilitate neural repair and regeneration, thus presenting a therapeutic potential that requires further investigation.

In summary, the present study indicates that hfNCSC-sEVs inhibit the expression of SMAD7 and activate the TGF-β/SMAD signaling pathway in PCs by delivering miR-21-5p. This mechanism results in HAS2 upregulation, thereby promoting PC proliferation and migration. Consequently, the expression of tight junction proteins in PCs is increased, which enhances their tubulogenic and barrier functions. Our findings reveal a novel mechanism for promoting PC regeneration and repair following peripheral nerve injury, thus offering new insights and directions for the clinical treatment of peripheral nerve injury.

## Data Availability

*No additional data are available*.
